# Junior Doctors’ Perceptions of Critical Care Education in Medical School and the Association with Confidence in Core Clinical Skills: A Survey of Graduates in Queensland, Australia

**DOI:** 10.1007/s40670-026-02797-2

**Published:** 2026-07-14

**Authors:** Sree Boddeti, Ishvar Nedunchezhian, Vinod Gopalan

**Affiliations:** 1 Medical Education, School of Medicine & Dentistry, Griffith University, Gold Coast Campus, 4222 Gold Coast, Australia; 2 School of Medicine & Dentistry, Griffith University, Gold Coast Campus, QLD 4222 Gold Coast, Australia

**Keywords:** Critical care education, Undergraduate medical education, Anaesthetics placement, Junior doctor preparedness, Clinical skills confidence, Intensive care unit placement

## Abstract

Confidence in critical care skills is essential for junior doctors. However, exposure to anaesthetics and critical care during medical school remains limited. This study evaluates how perceptions of undergraduate critical care teaching influence junior doctors’ confidence in core clinical skills. A cross-sectional survey was distributed to junior doctors across Queensland, Australia. Quantitative data captured exposure to anaesthetics, perceived quality of teaching, and confidence in skills such as bag-mask ventilation (BMV) and pre-anaesthetic assessment. Qualitative data explored factors influencing career interest in critical care. Quantitative analysis was performed using chi-square testing, and qualitative responses underwent thematic analysis. Sixty junior doctors completed the survey. Most reported 0–2 weeks of anaesthetic placement, and 48.3% rated their teaching as “intermediate.” Despite limited exposure, 61.7% reported confidence performing BMV, and 50% in pre-anaesthetic assessments. Higher perceived quality of teaching was significantly associated with greater confidence in BMV (*p* = 0.009) and pre-anaesthetic assessment (*p* = 0.008). Placement duration alone did not correlate with BMV confidence but did correlate with pre-anaesthetic assessment confidence (*p* = 0.004). Qualitative analysis identified themes relating to lifestyle, shift work, speciality exposure and competing interests in other specialties. l Perceived quality of undergraduate critical care education was associated with increased confidence in core skills. Placement duration alone may not improve confidence in high-risk procedures such as BMV. Implementing standardised learning objectives, structured placements, and earlier clinical exposure may improve preparedness, support safer clinical practice, and better inform career choices among junior doctors.

## Background

Anaesthesia and critical care are fundamental components of hospital medicine, with junior doctors frequently required to assess and stabilise critically unwell patients. The specialty of anaesthesia continues to expand, driven by increasing demand for peri-operative and critical care services, and is now one of the largest departments in many hospitals [1, 2]. Despite this growing importance, undergraduate medical curricula in Australia often provide limited theoretical teaching and minimal clinical exposure in anaesthesia and critical care. A survey of medical institutions demonstrated that Australasia had the lowest proportion (40%) of designated anaesthetic medical education departments internationally, highlighting a longstanding educational gap [3].

Core clinical skills such as cannulation, airway management, and pre-operative assessment are essential for junior doctors; however, exposure to these skills during medical school varies considerably. Contributing factors include reduced supervisor availability, inconsistent student engagement, and limited placement duration, particularly within anaesthesia rotations [4]. Strengthening training in these areas offers several benefits, including more confident junior doctors, improved patient safety, and increased recruitment into anaesthesia and critical care specialties [5, 6].

Although the adequacy of critical care education has been discussed for more than a decade, there has been little substantive change in undergraduate curricula in Australia or internationally. Notably, few studies have examined the perspectives of junior doctors—those who most directly experience the transition from medical student to clinical practice. Their insights are essential to determining whether current teaching effectively prepares graduates for real-world responsibilities.

Existing evidence suggests persistent gaps in critical care exposure, and limited research exists on the junior doctor’s perception of this exposure in Australia. A 2012 survey reported that nearly one-quarter of Australian and New Zealand medical programmes offered no mandatory or optional intensive care unit (ICU) placement, and among those that did, placement duration was typically less than one week [7]. In international studies looking directly at perspectives of junior doctors: first-year graduates in the United Kingdom reported feeling underprepared for managing acutely unwell patients due to insufficient undergraduate exposure [8], while American studies have shown that medical students with greater ICU experience demonstrated markedly improved confidence in recognising critical illness during residency [9]. Importantly, targeted interventions—including enhanced critical care placements and structured educational courses—have consistently been shown to improve student confidence and competence [10, 11].

Early exposure may also confer additional benefits. Studies show that critical care teaching introduced during pre-clinical years is both effective and well-received, particularly when delivered through face-to-face clinical interactions [12, 13]. Placement experience not only improves competency but may also encourage interest in pursuing these subspecialty careers [14].

More broadly, outside of critical care, surveys have shown that despite being generally satisfied with the medical training provided, junior doctors often felt a lack of clinical relevance of content and insufficient competency in procedural skills. Typically stemming from limited clinical teaching and practical exposure during training [15, 16]. However, these surveys looked generally at medical training instead of focusing on any one field.

Despite these insights, little research has specifically explored how Australian junior doctors perceive the adequacy of their undergraduate critical care education and how this relates to their confidence in essential clinical skills. Addressing this gap is crucial for evaluating whether current curricula are aligned with the realities of junior medical practice and for identifying opportunities to strengthen training.

The framework underpinning this study is provided by the experiential theory of learning [17]. This theory proposes that learning is enhanced through experience, active participation in clinical settings and reflection. Particularly, whether the use of experiential learning in conjunction with traditional didactic methods may influence the confidence of learners and enhance their perceived preparedness for practice in critical care. The primary aim of this study was to evaluate junior doctors’ perceptions of the adequacy of anaesthetic and critical care teaching during medical school and to examine how this relates to their confidence in core clinical skills. We hypothesised that junior doctors who perceived their undergraduate critical care teaching as more adequate would report greater confidence in key critical care skills. We further hypothesised that greater clinical exposure, measured by anaesthetic placement duration, would also be associated with higher confidence in these skills. A secondary aim was to explore factors influencing their interest in pursuing a career in critical care. Given the studied literature demonstrating the influence of clinical exposure on career choice. We hypothesised that respondents would identify exposure-related and lifestyle-related factors as important determinants of career interest.

## Methods

### Study Design and Data Collection

A descriptive cross-sectional survey was conducted to evaluate junior doctors’ perceptions of their undergraduate anaesthetic and critical care education. The study was considered cross-sectional because the data collected provided a snapshot of participants’ perception of received education and conferred self-reported confidence shortly after graduating. As individuals progress through their medical career, there are numerous influences on the measured variables in this study, so a longitudinal approach was not favoured. The survey was designed to collect predominantly quantitative data with a small qualitative component. The survey was informed by experiential learning theory, with survey items specifically designed to explore relationships between clinical exposure and perceived quality of educational experience and confidence in critical care skills. A preliminary literature review using PubMed (search terms: “anaesthetic medical education,” “anaesthetic education in medical school,” “anaesthesia student”; restricted to the past 25 years) was undertaken to identify existing gaps in the field and to guide survey development.

A survey methodology was selected as the study sought to capture perspectives from junior doctors across multiple hospitals in Queensland. Surveys facilitate the efficient collection of both quantitative and qualitative data despite the geographical dispersion of participants, whilst maintaining anonymity. Other methodologies such as interviews and focus groups were considered; however would be significantly more resource-intensive, confer a smaller sample size, and reduce participant anonymity.

Sixty eligible participants were junior doctors within one to three years post-graduation. No exclusion criteria were applied. The survey was administered using Microsoft Forms and distributed via closed online social media groups accessible only to junior doctors in Queensland, Australia. Participation was voluntary and anonymous. Study information and consent were embedded at the start of the survey. Given the low-risk nature of the research, no foreseeable risks to participants were identified.

This study was approved by the Griffith University Human Research Ethics Committee (GU Ref No: 2024/819). All participants provided informed consent prior to participation.

## Survey Development and Validation

The survey was developed to look at critical care education in medical schools. Firstly, observing the face validity of the survey. It broadly covers a mixture of confidence in critical care skills and knowledge. Additionally, it explores the perceived adequacy of medical education in this topic, which is the crux of this paper’s objective. Bag-Mask ventilation was selected because it is a fundamental airway management skill and a critical component to managing deteriorating patients. It is commonly taught in life-support modules of medical curricula and frequently encountered in critical care settings [18]. A pre-anaesthetic assessment was chosen as the “history” component, as this is also often a safe practice for students to engage in for all patients during their anaesthetic time. Similar to above, the ability to do a basic perioperative review of a patient is pertinent to every junior medical doctor. Specific content validation was ensured through consulting anaesthetists and current medical educators who reviewed the survey to allow for clinical and academic relevance. Revisions were made accordingly. The survey consisted of closed-ended (multiple choice, Likert scale); in addition to one open-ended question, which aimed to ascertain the reasons for which individuals pursued or avoided a critical care career. The initial survey had questions on a 5-point Likert Scale (e.g., Very confident, somewhat confident, neutral, not very confident, no confidence). For the purposes of analysis, the results were condensed to a 3-point scale (e.g. confident, neutral, minimal confidence). This was done to reduce the sparsely populated cells and improve the validity of the Chi-Square testing. Neutral responses remained unchanged. This same approach was applied to other 5-point Likert scale variables across the study.

Most survey variables were measured using single-item questions rather than multi-item scales. As such, formal psychometric testing (e.g., factor analysis, Cronbach’s alpha) was not performed, which is acknowledged as a potential limitation.

### Data Analysis

Descriptive statistics were used to summarise participant demographics, including the year of graduation, university attended and any previous exposure in critical care. Specific to the study objectives, the following parameters were collected regarding participants’ medical school experience: perceived adequacy of critical care education in medical school, critical care placement preparedness and placement duration. The parameters used to investigate junior doctor confidence included confidence in bag-mask ventilation, pre-anaesthetic evaluation, and general critical care knowledge base.

Quantitative data were analysed using IBM SPSS Statistics (Version 31). Specifically, we examined relationships between perceived teaching quality (independent variable) and self-reported confidence in BMV and pre-anaesthetic assessment (dependent variables). We also tested whether anaesthetic placement duration (independent variable) was associated with confidence in the same clinical skills. These skills were selected as they safely fall within the scope of junior doctors or medical students and were used as a snapshot tool to assess their critical care skill and knowledge confidence.

Pearson’s chi-square tests were used to assess associations between various parameters, of which the statistically significant associations have been further discussed in this paper. Primarily, the perceived teaching quality, anaesthetic placement time and self-reported confidence in specific skills (BMV and pre-anaesthetic assessment) were analysed. Statistical significance was set at *p* < 0.05. Assumptions of minimum expected cell counts were tested prior to application.

Qualitative free-text responses were analysed using thematic analysis described by Braun & Clarke [19]. Investigators independently reviewed responses and familiarised themself with the data before identifying recurring themes, words, concepts, and patterns. Initial codes were generated independently and subsequently discussed to reach consensus on overarching themes. These themes included ‘lifestyle’, ‘salary’ and ‘exposure’. Independent coding and consensus discussions were used to enhance the trustworthiness and credibility of the qualitative findings.

## Results

### Participant Demographics

**Fig. 1:**
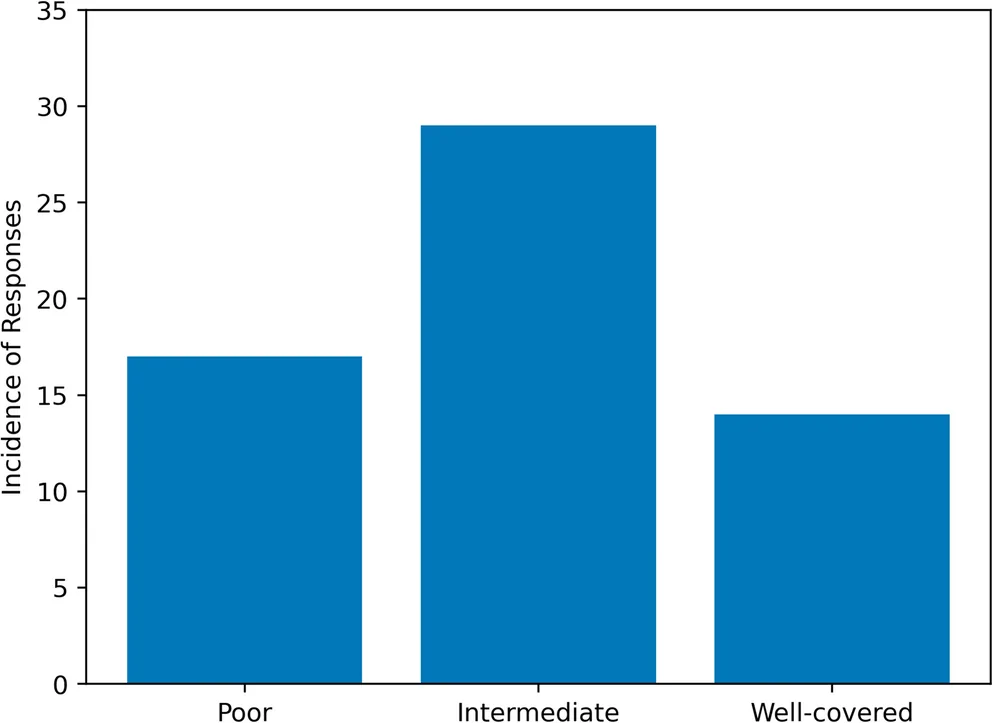
Perceived adequacy of medical school critical care education. Distribution of responses categorised as poor, intermediate, or well-covered

**Fig. 2:**
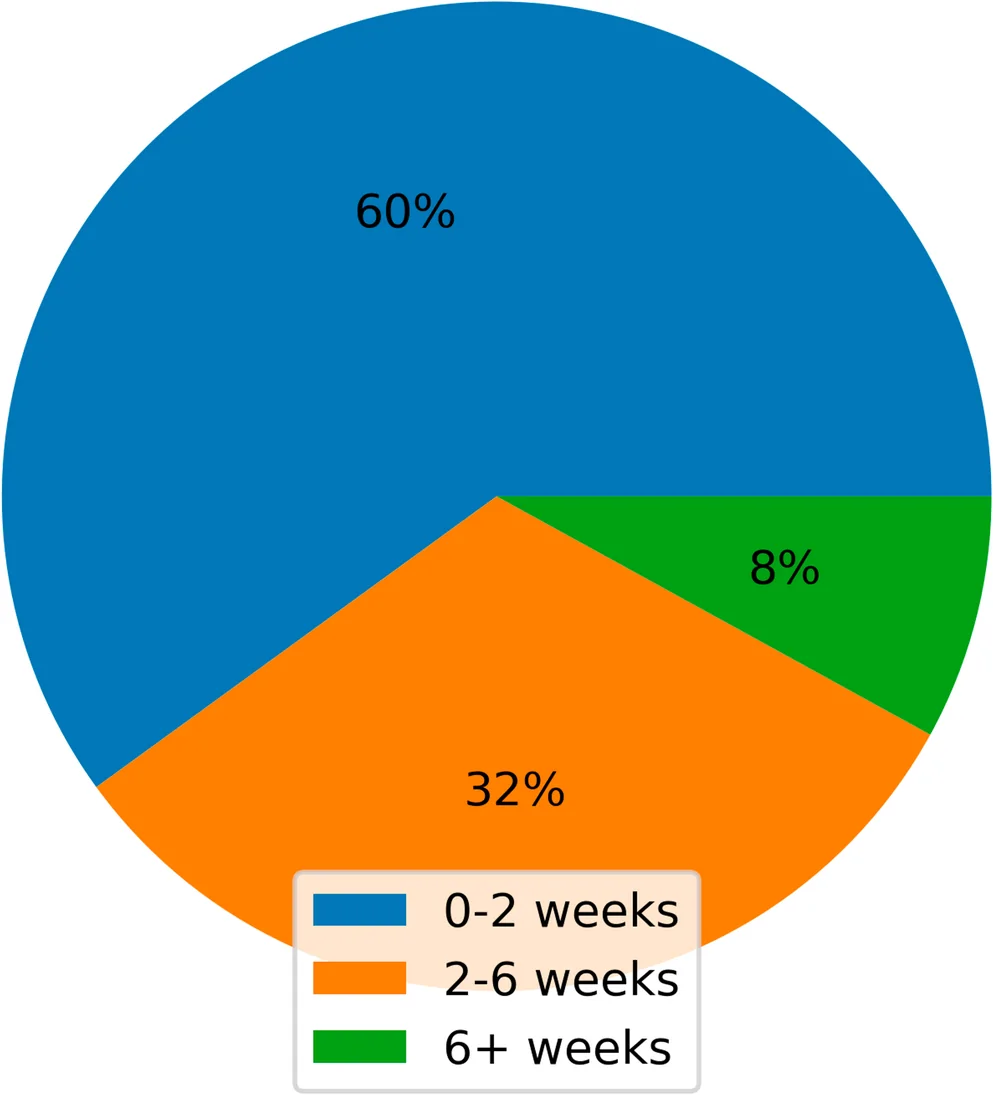
Distribution of anaesthetic placement durations among respondents. Pie chart illustrating the proportion of students reporting various durations of anaesthetic placements

A total of 60 junior doctors completed the survey, representing graduates from multiple medical schools across Queensland, Australia. Nearly half (48.3%, *n* = 29) rated their anaesthetic teaching during medical school as “intermediate”, while only 23% (*n* = 14) considered it “well covered” (Fig. 1). Most respondents (60%, *n* = 36) reported 0–2 weeks of anaesthetic placement experience (Fig. 2). Overall, 87% (*n* = 52) felt their critical care preparedness at graduation was either borderline (47%, *n* = 28) or sufficient (40%, *n* = 24).

More than half of the participants (61.7%, *n* = 37) reported feeling confident in performing bag-mask ventilation (BMV). Confidence in completing a pre-anaesthetic assessment was lower, with only 10% (*n* = 6) reporting clear confidence, 50% (*n* = 30) reporting neutral confidence, and 40% (*n* = 24) reporting a lack of confidence (Table 1).

**Table 1:** Perceived confidence in selected critical care skills

Confidence level	Bag–mask ventilation	Pre-anaesthetic assessment
Not Confident	9	24
Neutral	14	30
Confident	37	6

### Quantitative Associations

The first study hypothesis states that enhanced perception of teaching quality in medical school relates to an increased confidence in critical care skills, which was supported by statistically significant data. Junior doctors who rated their anaesthetic teaching as adequate were more likely to report confidence in both pre-anaesthetic evaluation (*p* = 0.008) and BMV (*p* = 0.009).

The second hypothesis states that increased clinical exposure to critical care is related to a general increase in critical care skill confidence. This was only partially supported by the data, where placement duration showed mixed associations. Increased anaesthetic placement time (2–6 weeks) was not associated with higher preparedness for critical care nor with greater confidence in BMV. However, a significant association was identified between longer placement duration and increased confidence in pre-anaesthetic assessment (*p* = 0.004). This likely reflects that pre-anaesthetic assessment is a task commonly accessible to medical students, with confidence improving through repeated real-world exposure.

## Qualitative Findings – Thematic Analysis

The qualitative analysis identified four overarching themes which influence career interest in critical care: perceived work-life balance, exposure to the speciality, concerns regarding shift work and pre-existing interest in alternative specialities. Amongst participants expressing interest in critical care (32%, *n* = 20), the most reported theme was the perceived lifestyle and work-life balance offered by the speciality.

Respondents who were unlikely to pursue a career in critical care (54%, *n* = 32), cited the demands of shift work and a stronger interest in another speciality. A small number specifically reported that limited exposure in medical school was a barrier to considering a career in critical care.

## Discussion

This study examined junior doctors’ perceptions of their preparedness for critical care and confidence in key anaesthetic-related skills, within the context of their undergraduate medical education in Queensland, Australia. As most participants completed medical training within the state, the findings likely reflect shared curricular structures and teaching models across institutions.

### Perception of Critical Care Education and Confidence

Only a small proportion of respondents (23%, *n* = 14) reported that their critical care teaching was “well-covered,” with the majority rating it as “intermediate” or “poorly covered.” Given the importance of critical care exposure, these findings suggest a need for more robust opportunities for medical students in this domain. This limited critical care exposure has been recognised as a key area of curriculum development [20].

A key observation was that doctors who perceived their anaesthetic and critical care teaching as adequate reported significantly greater confidence in both bag-mask ventilation (BMV) and pre-anaesthetic assessment. This suggests that *perceived* quality of undergraduate teaching may positively influence self-assessed preparedness in critical care. Several factors may contribute to this association. Firstly, those who felt teaching in medical school was adequate may have been more engaged in the didactic resources provided (i.e. lectures, modules) and taken the time to utilise self-directed learning [21]. Similarly, those same students are likely to have been more engaged in placements, attendance and opportunities. These factors would increase the level of confidence in critical thinking through sheer engagement, compared to their counterparts who did not actively seek out resources or opportunities [22, 23].

Supervisor-dependent factors also play a role. Those who had positive experiences with their critical care teaching may have had engaging and supportive teachers in this space. This will not only enhance their perception of education quality but is likely to increase their confidence due to better teaching and opportunity availability [24]. For example, a supervisor who is constantly teaching during an anaesthetic case and allows students to bag-mask ventilate, practice line insertions, nuance their history taking skills, etc. Variable teaching styles and attitudes across supervisors lead to inconsistent experiences, which further contribute to disparities in confidence and preparedness among graduates [25]. Medical schools may consider formal supervisor training and standardised learning objectives to promote more consistent student experiences [20, 26].

It is important to note that perceptions of teaching adequacy and confidence are inherently subjective and do not necessarily reflect actual competency. Notably, over 60% of respondents reported confidence in performing BMV despite minimal exposure. This illustrates the well-documented phenomenon of the “illusion of competence” in medicine, in which learners overestimate their ability due to limited experience or feedback [27]. Such overconfidence may pose risks in high-stakes clinical settings if not addressed through structured training and supervision [28–30].

### Placement Duration

Interestingly, placement duration alone did not significantly impact confidence in BMV or overall preparedness, suggesting that the quality and structure of clinical exposure may be more influential than time spent [31]. However, longer placement duration (2–6 weeks) was associated with increased confidence in pre-anaesthetic assessments, a skill more likely to be practised autonomously and repeatedly during student placements. This distinction supports the importance of real-life, hands-on learning opportunities, particularly for non-invasive or low-risk skills [32]. A small subset of respondents had prior post-graduate critical care exposure, which may have influenced their confidence levels [33]. However, as only 5% reported significant experience, this is unlikely to have substantially affected the overall findings.

### Qualitative Results – Future Careers in Critical Care

The study also explored junior doctors’ attitudes toward careers in critical care. Approximately one-third of respondents (*n* = 20) expressed interest in pursuing this field, often citing work–life balance as a key appeal. This is a common motivator for medical students [34, 35]. While critical care roles can offer long-term flexibility, this perception may not fully reflect the realities of prolonged training, shift work, and the demands of certain subspecialties [36]. This underscores the importance of increased clinical immersion to help students and junior doctors make informed career decisions [37].

Conversely, the most common deterrents included shift work, interest in other specialties, and limited undergraduate exposure. All of which are previously reported issues [38, 39]. These findings reinforce the notion that early exposure to critical care is likely integral in shaping career choices [20].

### Limitations

This study has several limitations. Firstly, surveys are limited by response bias, inability to clarify participant responses and a lower response rate compared to other qualitative approaches. Self-reported confidence may not reflect true competency, particularly for complex skills such as BMV. Recall bias is possible, as participants were at different stages post-graduation, and postgraduate rotations may have influenced confidence independently of undergraduate training. Selection bias may have occurred, with those interested in critical care more likely to respond. The study predominantly included graduates from Queensland universities, particularly Griffith University, which may limit generalisability. Future studies should examine whether these findings are replicated across medical schools with different curricula, including programmes with longer critical care placements. This would allow the results to be applied across diverse educational settings and healthcare systems. Lastly, qualitative findings are inherently subjective, and thematic analysis may be influenced by the investigator’s interpretation.

The findings of this study suggest that the perceived quality and structure of medical education in critical care are influential in conjunction with placement duration to shape junior doctors’ confidence. These findings support consideration of a more structured approach to critical care, including clearly defined learning objectives, consistent clinical experiences and opportunities for supervised skill development.

Although medical school curricula were not directly investigated in this study, creating state or national standards to deliver critical care education may help reduce variability in student experiences and preparedness. Early exposure to critical care environments may help bridge the gap between theoretical knowledge, practical skills, and professional confidence. Future research should evaluate specific educational interventions, such as simulation-based training, structured clinical placements and standardised curriculum, using objective measures of competence in conjunction with self-reported confidence. Addressing these educational gaps is essential not only for fostering safer clinical practice but also for enabling informed career decision-making.

## Conclusion

Critical care education remains an underrepresented component of many medical school curricula, with limited classroom-based teaching and clinical placement exposure available for students to develop key skills and gain insight into the specialty. Placement duration alone may not address this gap, as longer placements were not associated with greater BMV confidence. This suggests that whilst time in clinical environments can be helpful, the quality and structure of the learning experience is paramount. Ensuring students receive consistent, structured, supervised experiences with clear learning objectives and objective feedback may be more effective in developing confidence and competency in high-risk procedures.

Given that critically unwell patients are encountered across all areas of medicine and are often first managed by junior doctors, it is essential that medical schools equip graduates with both the confidence and competence to deliver safe and effective care. Strengthening critical care education is therefore vital not only for patient safety but also for developing a capable and prepared medical workforce.

## References

[1] Carlisle J, White S, Tobin A. The anaesthetist and peri-operative medicine: migration and evolution. Anaesthesia. 2016;71(S1):1–2. 10.1111/anae.13339 26620140

[2] Abbott T, Fowler A, Dobbs T, Harrison E, Gillies M, Pearse R. Frequency of surgical treatment and related hospital procedures in the UK: a national ecological study using hospital episode statistics. Br J Anaesth. 2017;119(2):249–57. 10.1093/bja/aex137 28854546

[3] Cheung V, Critchley L, Hazlett C, Wong E, Oh T. A survey of undergraduate teaching in anaesthesia. Anaesthesia. 1999;54(1):4–12. 10.1046/j.1365-2044.1999.00640.x 10209363

[4] Chuang Z. Anesthesiologists in the modern medical school curriculum: importance and opportunity. Can Med Educ J. 2023;14(2):175–7. 10.36834/cmej.76188 PMC10254111 37304636

[5] Dairi MS, Aljabri MK, Bahakim AK, Aljabri AA, Alharbi TA, Alsehli AH, et al. Knowledge and Competence Towards Critical Care Concepts Among Final Year Medical Students and Interns: A Cross-Sectional Study. Adv Med Educ Pract. 2022;13:1091–101. 10.2147/AMEP.S372119. PMC9507286 36157377

[6] Smith A, Sadler J, Carey C. Anaesthesia and the undergraduate medical curriculum. Br J Anaesth. 2018;121(5):993–6. 10.1016/j.bja.2018.06.002 30336870

[7] Buchman T, Draghia-Akli R, Adam S. Accelerating coronavirus disease 2019 therapeutic interventions and vaccines—selecting compounds for clinical evaluation in coronavirus disease 2019 clinical trials. Crit Care Med. 2021;49:1963–73. 10.1097/CCM.0000000000005295 PMC8507598 34495876

[8] Burridge S, Shanmugalingam T, Nawrozzadeh F, Leedham-Green K, Sharif A. A qualitative analysis of junior doctors’ journeys to preparedness in acute care. BMC Med Educ. 2020;20(1):12–12. 10.1186/s12909-020-1929-8 PMC6958569 31931784

[9] Lane-Fall M, Pascual J, Massa S. Critical care exposure and preparedness among internal medicine residents: results from the American College of Physicians In-Training Exam Survey. Am J Med. 2024;137(12):1276–81. 10.1016/j.amjmed.2024.08.017 PMC12590543 39197721

[10] Croley W, Rothenberg D. Education of trainees in the intensive care unit. Crit Care Med. 2007;35(2 Suppl):S117–21. 10.1097/01.CCM.0000252917.25301.18 17242600

[11] Quach B, King C. Integrated critical care curriculum for the third-year internal medicine clerkship. MedEdPORTAL. 2019;16:10874–10874. 10.15766/mep_2374-8265.11032 PMC7727608 33324745

[12] Al Ansari M, Al Bshabshe A, Al Otair H, Layqah L, Al-Roqi A, Masuadi E, et al. Knowledge and Confidence of Final-Year Medical Students Regarding Critical Care Core-Concepts, a Comparison between Problem-Based Learning and a Traditional Curriculum. J Med Educ Curric Dev. 2021;8:2382120521999669. 10.1177/2382120521999669. PMC8855474 35187261

[13] Luks A, King M. Early introduction to critical care medicine: impact on student knowledge and confidence. Med Educ. 2011;45(5):514–5. 10.1111/j.1365-2923.2011.03954.x 21486337

[14] Andrews GJ, Brodie DA, Andrews JP, Wong J, Thomas BG. Place(ment) matters: students’ clinical experiences and their preferences for first employers. Int Nurs Rev. 2005;52(2):142–53. 10.1111/j.1466-7657.2005.00262.x. 15842327

[15] Eyal L, Cohen R. Preparation for clinical practice: a survey of medical students’ and graduates’ perceptions of the effectiveness of their medical school curriculum. Med Teach. 2006;28(6):23. 10.1080/01421590600776578. 17074696

[16] Prince KJAH, Boshuizen HPA, Van Der Vleuten CPM, Scherpbier AJJA. Students’ opinions about their preparation for clinical practice. Med Educ. 2005;39(7):704–12. 10.1111/j.1365-2929.2005.02207.x. 15960791

[17] Department of Medical Education, School of Medical Sciences, Malaysia US, Kelantan, Malaysia, Ahmad A, Abd Rahim AF, Department of Medical Education, School of Medical Sciences, Universiti Sains Malaysia, Kelantan, Malaysia, Wan, Mohammad WMZ et al. Department of Community Medicine, School of Medical Sciences, Universiti Sains Malaysia, Kelantan, Malaysia,. A Scoping Review on Experiential Learning in Workplace-Based: Clinical Placements. Educ Med J. 2025;17(1):55–77. 10.21315/eimj2025.17.1.6

[18] Borremans G, Geerts A, Coppens M, Christiaens-Leysen E, Herbelet S, Van De Voorde P. Bag-mask ventilation by low- or untrained rescuers during adult and pediatric resuscitation: a randomized cross-over manikin study. Front Disaster Emerg Med. 2023;1:1274501. 10.3389/femer.2023.1274501.

[19] Ahmed SK, Mohammed RA, Nashwan AJ, Ibrahim RH, Abdalla AQ, Ameen M. Using thematic analysis in qualitative research. J Med Surg Public Health. 2025;6:100198. 10.1016/j.glmedi.2025.100198.

[20] Kim JL, Kim Y Intensive care unit-based medical education for medical students: a scoping review. Cureus. 2025;23. . 10.7759/cureus.88570. PMC12284912 40703327

[21] Cheng X, Ma XY, Luo C, Chen J, Wei W, Yang X. Examining the relationships between medical students’ preferred online instructional strategies, course difficulty level, learning performance, and effectiveness. Adv Physiol Educ. 2021;45(4):661–9. 10.1152/advan.00234.2020. PMC8435943 34498933

[22] Sadler HJ, John AA, Thomas S, Sevenoaks L, Medwell LJ, Viepadan A. Exploring Medical Student and Staff Perspectives on Clinical Placements: Factors Influencing Attendance and Engagement. Cureus. 2025. . 10.7759/cureus.93336 PMC12560193 41164050

[23] McNair R, Griffiths L, Reid K, Sloan H. Medical students developing confidence and patient centredness in diverse clinical settings: a longitudinal survey study. BMC Med Educ. 2016;16(1):176. 10.1186/s12909-016-0689-y. PMC4946086 27421655

[24] Keshavarzi MH, Azandehi SK, Koohestani HR, Baradaran HR, Hayat AA, Ghorbani AA. Exploration the role of a clinical supervisor to improve the professional skills of medical students: a content analysis study. BMC Med Educ. 2022;22(1):399. 10.1186/s12909-022-03473-w. PMC9128286 35606771

[25] Tasker F, Newbery N, Burr B, Goddard AF. Survey of core medical trainees in the United Kingdom 2013 – inconsistencies in training experience and competing with service demands. Clin Med. 2014;14(2):149–56. 10.7861/clinmedicine.14-2-149. PMC4953286 24715126

[26] Gergen DJ, Kommaraju K, Stewart NH, Shah NG, Neumeier AT. Reimagining Undergraduate Critical Care Medical Education: A Path for the Next Decade. Sch. 2024;5(3):375–85. 10.34197/ats-scholar.2023-0136PS. PMC11448824 39371228

[27] Rahmani M. Medical Trainees and the Dunning–Kruger Effect: When They Don’t Know What They Don’t Know. J Grad Med Educ. 2020;12(5):532–4. 10.4300/JGME-D-20-00134.1. PMC7594774 33149817

[28] Kovacs RJ, Lagarde M, Cairns J. Overconfident health workers provide lower quality healthcare. J Econ Psychol. 2020;76:102213. 10.1016/j.joep.2019.102213.

[29] Lighthall GK, Vazquez-Guillamet C. Understanding Decision Making in Critical Care. Clin Med Res. 2015;13(3–4):156–68. 10.3121/cmr.2015.1289. PMC4720506 26387708

[30] Beldhuis IE, Marapin RS, Jiang YY, De Simões NF, Georgiou A, Kaufmann T, et al. Cognitive biases, environmental, patient and personal factors associated with critical care decision making: A scoping review. J Crit Care. 2021;64:144–53. 10.1016/j.jcrc.2021.04.012. 33906103

[31] Shbeer A. Evaluating Student Satisfaction and Self-Confidence in Simulation-Based Anesthesiology Training among Final-Year Medical Students. Healthcare. 2024;12(15):1521. 10.3390/healthcare12151521. PMC11312120 39120224

[32] Taylor AWR, Anderson ES, Gay S. It’s a gamble’: A phenomenological exploration of medical students’ learning experiences as newcomers to clinical communities of practice. Clin Teach. 2024;21(4):e13708. 10.1111/tct.13708. 38058032

[33] Scott RW, Fredriksen K. Extracurricular work experience and its association with training and confidence in emergency medicine procedures among medical students: a cross-sectional study from a Norwegian medical school. BMJ Open. 2022;12(9):e057870. 10.1136/bmjopen-2021-057870. PMC9516200 36167389

[34] Asiri WMA, Shati AA, Alrowaibah NA, Althumairi RK, Alqahtani GM, Mahmood SE. The influencing factors of choosing future medical specialties among students in Saudi Arabia: A nationwide multicenter survey. Med (Baltim). 2023;102(14):e33483. 10.1097/MD.0000000000033483. PMC10082225 37026904

[35] Levaillant M, Levaillant L, Lerolle N, Vallet B, Hamel-Broza JF. Factors influencing medical students’ choice of specialization: A gender based systematic review. EClinicalMedicine. 2020;28:100589. 10.1016/j.eclinm.2020.100589. PMC7588859 33134904

[36] Okeke CC, Gboyega-Adejuwon AP, Zubair A, Okereke PU, Ikegwuonu OE, Anyamene EL, et al Perception of Medical Students on Anaesthesiology as a Specialty: A Systematic Review. Cureus. 2024. . 10.7759/cureus.73213. PMC11626989 39655135

[37] Schoon B, Kötter T. Intracurricular Factors Influencing Medical Students’ Specialty Choice: A Systematic Review. Adv Med Educ Pract. 2024;15:1127–40. 10.2147/AMEP.S491008. PMC11605303 39620065

[38] Chan DM, Wong R, Runnels S, Muhizi E, McClain CD. Factors Influencing the Choice of Anesthesia as a Career by Undergraduates of the University of Rwanda. Anesth Analg. 2016;123(2):481–7. 10.1213/ANE.0000000000001433. 27308955

[39] Gqiba A, Gopalan P, Evans C. Perceptions of final-year UKZN medical students about anaesthesia as a specialty choice. South Afr J Anaesth Analg. 2017;23(3):72–5. 10.1080/22201181.2017.1321862.

